# A Clinical Diagnosis of Wiskott Aldrich Syndrome in an Ethiopian Boy with Recurrent Sinopulmonary Infections: A Case Report

**DOI:** 10.4314/ejhs.v30i6.26

**Published:** 2020-11

**Authors:** Solomie Jebessa Deribssa, Tinsae Alemayehu

**Affiliations:** 1 St. Paul's Hospital and Millennium Medical College, Addis Ababa, Ethiopia; 2 American Medical Center, Specialty Clinic for Infectious, Diseases and Travel Medicine Addis Ababa, Ethiopia

**Keywords:** Child, Ethiopia, Primary immunodeficiency, Wiskott Aldrich syndrome

## Abstract

**Background:**

Wiskott Aldrich syndrome is a primary immunodeficiency notable for eczema, recurrent infections, bleeding diathesis and microcytic thrombocytopenia.

**Case:**

A 4½ year old boy presented with recurrent sinopulmonary infections, repeated treatment for severe eczema since infancy, thrombocytopenia with low platelet volume. His brother and uncles died during childhood due to repeated illnesses. We outline ways to diagnose and manage children in resource limited settings.

**Conclusion:**

Wiskott Aldrich syndrome can be diagnosed by its clinical triad of syndromes. Mutation of the WASP gene confirms diagnosis. Increasing reports of primary immune deficiencies in Ethiopia call for improved education and care for clinical immunology.

## Introduction

Combined immune deficiencies with syndromic features account for 13% of Primary Immune Deficiencies (PIDs). Notable members include Wiskott Aldrich Syndrome (WAS), Ataxia-Telagiectasia and Hyper immunoglobulin E syndrome (1).

Wiskott Aldrich syndrome (WAS) is an X-linked recessive disorder diagnosed in 1 in 100,000 live births and which affects inginnate and acquired immunities. A mutation in the WASp gene in hematopoietic cells impairs assembly of actin filaments required for cell migration and cell-cell interaction. This leads to impaired functions of T-cells, B-cells, NK cells and platelets and predisposes affected males for small numbers of small platelets, severe eczema, hematogenous malignancies and autoimmune disorders (2).

WAS was first identified in 1937 by Dr Alfred Wiskott and its inheritance confirmed by Dr Robert Aldrich (3). We summarize the presentation of a 4 years and 6 months old Ethiopian boy with features of WAS. We also outline how the diagnosis can be reached in resource limited settings and options for care.

## Case Presentation

We report on a 4 years and 6 months old Ethiopian boy who presented to Tikur Anbessa Specialized Hospital, Addis Ababa, with a recurrence of cough and fast breathing of five days. His respiratory complaints had been waxing and waning over the past few months and was at presentation on his 5^th^ month of anti-tuberculous treatment which was not relieving his illness. He also had low grade fever, grunting, poor appetite and failure to thrive. His past medical history showed repeated treatment for pneumonia and otitis media. He also had epistaxis and occasional passage of stool mixed with bright red blood since infancy. He was on follow-up for generalized itchy skin lesions since 7 months of age. He was treated at age 2 years for pulmonary tuberculosis and declared cured.

He was the third child for his family, a product of non-consanguineous parents. His immediate older brother had died at age 2 years of similar illness. He has an apparently healthy older sister. All maternal brothers had died in childhood due to recurrent severe infections. There was no history of delayed separation of his umbilical cord. He received all ageappropriate vaccines with no post-vaccine adverse reactions reported. Despite not being vaccinated against meningococcal infections, he had record of being treated for neither meningococcemia nor meningitis.

**Figure 1:**
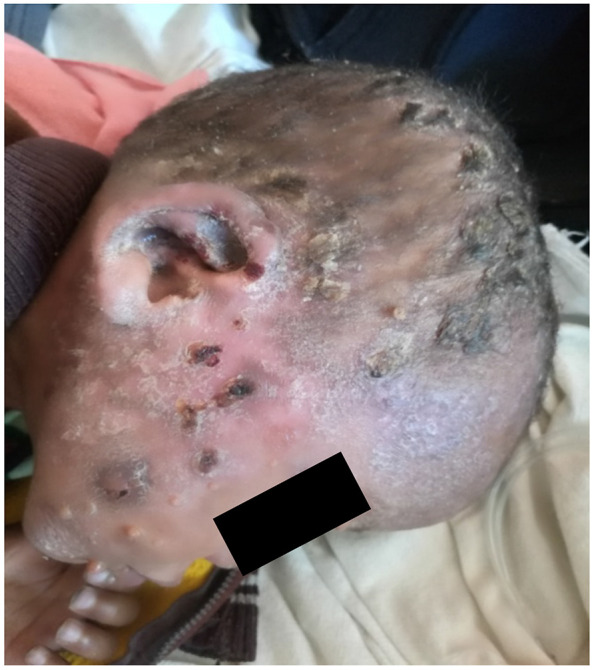
Crusted lesions on scalp and face and Pinna

**Figure 2:**
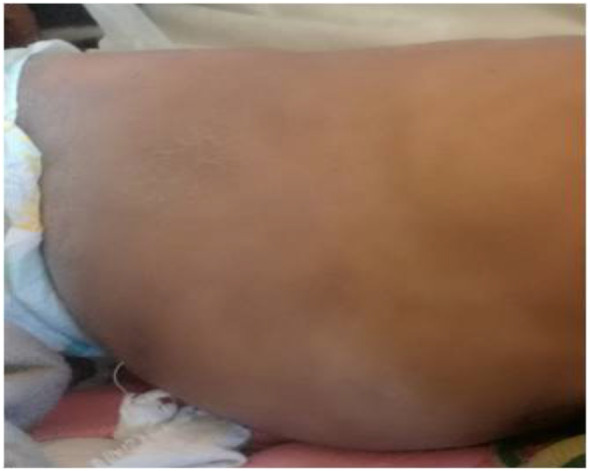
Healed hypo-and hyperpigmented, scaly lesions on the trunk and extremities)

He appeared chronically sick with extensive skin lesions but no dysmorphia. His pulse rate was 108/minute, respiratory rate 52/minute, temperature 36.4oC and saturation of oxygen in room air of 82%. He was underweight (weight = 12 kg). He had profuse purulent discharge from his right ear with an excoriated right pinna. He had no oral thrush. He had submandibular lymphadenopathy and hepatomegaly of 12 cm. He had crusted lesions on the scalp and face (Figure 1) and hypopigmented lesions on trunk and extremities (Figure 2). He also hadumblicated small nodules over face and extremities and clubbing.

His total white blood cell was 6800/mm^3^ (normal for age: 4000 – 12000/mm^3^). His absolute neutrophil count was 3700/mm^3^, absolute lymphocyte count was 2300/mm^3^, absolute eosinophil count was 14/mm^3^.

His hemoglobin was 10.2 g/dl, mean corpuscular volume 81.4 fl, platelets 9000/mm^3^ (normal: 150,000 – 400,000/mm^3^) and mean platelet volume 8.5 fl (normal 9.4 – 12.3 fl).

The peripheral smear showed thrombocytopenia with no inclusions in lymphocytes, neutrophils or platelets. His chest X-ray showed bilateral reticulonodular infiltrates and no thymic aplasia. A GeneXpert molecular test from his gastric aspirate was negative for *Mycobacterium tuberculosis*. His abdominal ultrasound showed hepatomegaly with no focal lesions. His HIV serologic test was negative. His short hospital stay could not enable work-up for autoimmune disorders.

A clinical diagnosis of WAS was made based on his gender, early death of male family members, recurrent pulmonary and ear infections, bleeding tendencies, eczema, thrombocytopenia, small platelets, normal lymphocyte and neutrophil counts. Despite parenteral and otic antibiotics and platelet transfusions, the platelet count remained below 20,000/mm^3^ and he succumbed due to his illness. Prophylactic Trimethoprim-Sulfamethoxazole and Acyclovir had been initiated. Intravenous immunoglobulins were not available in the hospital formulary. Hematopoietic cell transplantation (definitive treatment) is not performed in Ethiopia.

## Discussion

Wiskott-aldrich syndrome consists of a triad of eczema, recurrent infections and bleeding diathesis (small numbers of small platelets). The classic triad occurs in 1/3^rd^ of cases. While a third of cases can occur de novo, the majority have an X-linked recessive inheritance. While serum immunoglobulins G and M are low, levels of immunoglobulins, A and E are high (2).

A predominantly antibody deficiency presents after six months of life and is notable for small or absent lymph nodes and tonsils. Non-syndromic PIDs affecting both humoral and cellular arms of immunity present in the first three months of life with lymphopenia (1,4). Both are unlikely considering our patient's physical examination and normal white blood cell counts.

Syndromic primary immune deficiencies like WAS have unique presentation: Leukocyte adhesion defect (Delayed umbilical cord separation and marked leukocytosis), Ataxiatelangiectasia (Oculocutaneoustelangiectasias and truncal ataxia), Chediak-Higashi syndrome (Oculocutaneous albinism and giant lysosomal inclusions) and Hyperimmunoglobulin E and Omenn syndromes (Eosinophilia) (4). Complement disorders predispose for recurrent meningococcal infections and autoimmune disorders.

Our patient's presentation and his and his male family members' affliction make the most likely X-linked PID. Among X-linked PIDs, X-linked severe combined immune deficiencies were ruled out by normal lymphocyte counts and late presentation. X-linked agammaglobulinemia and hyperimmunoglobulin M syndrome are predominantly antibody deficiencies with absent or small lymph nodes. Microthrombocytopenia is not observed in chronic granulomatous disease (2,4).

We could not do genetic confirmation for WAS. The treatment consists of hematopoietic stem cell transplantation and platelet transfusions. Recurrence of infections can be prevented by Trimethoprim-Sulfamethoxazole, Acyclovir and intravenous immunoglobulin (IVIG). Live attenuated vaccines are contraindicated (5).

In conclusion, we report an Ethiopian boy with the classic triad of recurrent sinopulmonary infections, eczema and microthrombocytopenia of the X-linked primary immunodeficiency, Wiskott Aldrich syndrome. Early recognition and referral enhance survival. Increasing reports of PIDs in Ethiopia call for improved education and care for clinical immunology.

## References

[1] Erjaee A, Bagherpour M, van Rooyen C, van den Berg S, Kinnear CJ, Green RJ (2019). Primary immunodeficiency in Africa – a review. S Afr Med J.

[2] Galal N, Ohida M, Meshaal S, AbdElAziz D, Elhawary I (2019). Targeted screening for primary immunodeficiency disorders in the neonatal period and early infancy. Afri Health Sci.

[3] Aldrich RA, Steinberg AG, Campbell DC (1954). Pedigree demonstrating a sex-linked recessive condition characterized by draining ears, eczematoid dermatitis and bloody diarrhea. Pediatrics.

[4] Eley B, Esser M (2014). Investigation and management of primary immunodeficiency in South African children. SAfr Med J.

[5] Buchbinder D, Nugent D J, Fillipovich A H (2014). Wiskott-Aldrich syndrome: diagnosis, current management, and emerging treatments. The application of clinical genetics.

